# Innovation and financial sustainability in China's long-term care insurance: An empirical analysis of the Nantong pilot

**DOI:** 10.3389/fpubh.2022.1024491

**Published:** 2022-11-25

**Authors:** Peng Guo, Yajun Li

**Affiliations:** ^1^Department of Public Administration, China University of Labor Relations, Beijing, China; ^2^Department of Public Administration, Shandong Technology and Business University, Yantai, China

**Keywords:** long-term care insurance, long-term care service, financial sustainability, full coverage, path breaking, non-differentiated system

## Abstract

**Background:**

Against a backdrop of aging and declining birthrates, the demand for long-term care insurance (LTCI) systems in various countries throughout the world continues to rise. Most traditional LTCI systems only cover a limited group of people, which cannot solve the problem of nursing needs among other groups, and, further, it makes financial sustainability very difficult to achieve.

**Purpose:**

This article aims to explore how Nantong successfully achieves both “full coverage” and “financial sustainability” through institutional innovation.

**Objects:**

Institutional innovation logic and financial sustainability of LTCI system in Nantong, one prefecture-level city with the highest degree of aging in China.

**Methods:**

Through field interviews and research, this article examines the LTCI system in Nantong, exploring its logic and its successful realization of institutional innovation, which combines international and local experience. The study further uses quantitative methods to measure the system's financial sustainability.

**Data:**

From the seventh national population census bulletin, the 13th Five-Year population development plan, the Nantong Municipal Bureau of Statistics and Nantong Statistical Yearbook 2020 from Nantong government. And from the authors' field survey data in the Medical Insurance Bureau of Nantong.

**Results:**

The findings reveal that the Nantong model effectively disperses institutional risks by means of horizontal and vertical transfer payments, diversified financing, and service outsourcing, achieving its dual goals of full coverage and financial sustainability in the long run.

**Conclusion:**

Nantong Model has interrupted the path dependence of traditional dual systems and the philosophy of fragmented institutional construction. Nantong's path-breaking institutional construction paradigm has important theoretical and practical value.

**Contributions:**

The experience of Nantong may prove valuable and instructive, for not only other cities in China but also developing countries across the globe.

## Introduction

With the deepening of population aging, some countries have established long-term care insurance (LTCI) systems, and some are considering or studying how to establish such systems. Developed countries that have established LTCI systems, such as Germany and Japan, have relatively high levels of economic development and sound welfare systems. Their institutional models are often difficult for other developing or less-developed countries to emulate. As the world's most populous superpower, China's population aging problem is very serious, and the problem of “growing old before getting rich” is prominent. Research on China's LTCI systems can serve as an important reference for low- and middle-income countries. However, unlike Western countries, due to the substantive differences among Chinese cities in terms of financial situation, economic development, and population aging, reforms in the field of social policy are often piloted by one city and then promoted in others. In short, while social policy reform in Western countries follow a “legislation first” model, China utilizes a “pilot first” approach. Since China has not yet promulgated national policies and laws in the field of LTCI, the pilot program is likely to become the policy blueprint for promoting an LTCI system in the future. Therefore, it is crucial to study the methods and implementation effects for LTCI systems in pilot cities.

The Nantong Model was introduced earlier than the first batch of pilot policies in China, it has provided an extremely important reference for the first and second batch of pilot cities, and may even become a blueprint for China's promotion of the LTCI system in the future. Therefore, the study of Nantong model has important theoretical and practical significance.

Among the first to become a pilot city for LTCI, Nantong is known as the hometown of longevity in China. According to the international classification standard, when the population aged 65 and above accounts for 14% or more of the population, a country or region becomes a deeply aging society. Nantong attained this status as early as 1983, 17 years ahead of China's average.^1^ According to data from the seventh census of the National Bureau of Statistics of China, conducted in 2021, 149 cities in China have become deeply aging societies. Among them, Nantong ranks first, with the highest degree of aging. The 2021 census revealed that 30.1% of the population were aged 60 and over, and 22.67% were aged 65 and over, considerably higher than the national level of 18.70 and 13.50%, respectively.^2^ In addition, Nantong had implemented family planning policy very early on, a typical example of national policy and low birthrates. In 1972, Nantong took the lead in realizing the plan of the State Council to reduce the natural growth rate to less than 10%, 8 years ahead of schedule and 26 years ahead of China's attainment of this goal. In 1990, although the country was in the third peak of population increases through births, the fertility level of Nantong's population had dropped to near the replacement level, and by 2000, it had dropped rapidly, to 1.22. By the sixth census in 2010, the total fertility rate in Nantong had dropped to 0.95, just half of the level advocated by the state. Even under such unfavorable demographic conditions, Nantong has carried out institutional innovation, and successfully established China's first full coverage LTCI system, which has achieved remarkable results (1). Although Nantong is the most profoundly aging prefecture-level city in China, its performance in the first series of pilot cities has been outstanding. Through institutional innovation and the comprehensive use of both horizontal and vertical transfer payments, it not only breaks from the path of traditional dual systems path and fragmented system construction, namely, “creating different systems for different groups of people,” but also achieves the dual goals of “full coverage” and “financial sustainability.” This achievement thus has important theoretical and practical value. The learning of its institutional concepts has spread to other pilot cities. For example, the policy of universal coverage to dilute the payment burden has been embraced by Suzhou, Jingmen, Shangrao, and Shihezi. The policy of including moderate disability in the scope of reimbursement to expand benefits and enhance payment incentives has been implemented in Suzhou, Guangzhou, and other cities. Home care cash subsidies to encourage family care policies have been deployed by Anqing, Chengde, Shangrao, and Shihezi (2).

This study utilizes mixed methods to explore the case of Nantong. It first employs qualitative research methods to analyze the institutional logic behind the city's LTCI reform through field interviews with members of relevant departments involved in the reform. Based on these interviews, this article elaborates the innovation-driving mechanisms and system design characteristics of Nantong's LTCI system. It analyzes the internal relationship between financial balance and design innovation, summarizes the advantages, experiences, and existing problems of the Nantong model, and provides generalizable experience and inspiration for the establishment of LTCI systems in similar regions.

Upon this conceptual foundation, the study conducts a quantitative analysis on the financial sustainability of the LTCI system in Nantong, which represents the most important goal in the design of the social insurance system and the most affected by the rapid aging and declining birthrates. This article elaborates the relevant parameters and builds models on the basis of officially released data and data obtained through surveys, to examine the long-term financial sustainability of the Nantong model.

## Literature review

LTCI is essentially a type of social insurance, a dispersal and financing mechanism for disability risks (3). Achieving long-term financial sustainability is a key objective and basic guideline for the design of all social insurance systems. Aging and an increase in life expectancy represent two factors precipitating unsustainable financial pressure. Aging leads to a decline in the number of contributors and an increase in the number of beneficiaries, and increased life expectancy means longer benefit payout periods (4, 5). The primary goal of LTCI reform is to reduce the impact of population aging through policy innovation. From the perspective of insurance participation and payments, it is necessary to control current payments to match the level of potential payment willingness. The main policy innovation is to extend the cost allocation time as much as possible and expand the scope of risk diversification (6, 7). With regard to treatment and payment, by optimizing the structure of different payment methods (8, 9), the goal is to cultivate service providers such as families, communities, and institutions, and to improve the quantity and efficiency of service supply (10).

A major problem faced by LTCI in the process of expanding coverage is how to overcome the problem of low willingness to participate caused by “low risk incidence rates but huge expenditure amounts”. The demand for cost sharing through coverage expanding is high, but the motivation to participate in LTCI is lacking. Lipszyc et al. analyzed data on the deaths of 80,000 elderly people and found that “short-term emergency treatment costs” were not significantly related to longevity, but “end-of-life care costs” increased substantially with lifespan. Nursing expenses for a 70-year-old deceased individual accounted for 10% of total medical expenses, while the nursing expenses for a 90-year-old individual accounted for 36% (11). At the same time, disability risk has the characteristics of a long and “thick” tail. Even for high-income families, long-term care expenditure accounts for 60% of personal disposable income, and for lower-income families, it is catastrophic (12, 13). However, unlike the risk of disease, the probability of disability is relatively low. People are thus more susceptible to irrational decision-making behaviors such as short-sightedness, loss aversion, and the Narrow Framing Effect, which lead to institutional impacts on the population. Risk coverage is insufficient (14, 15).

Another problem LTCI facing is the insured generally requires physical care services rather than cash, and is highly dependent on physical payments. The cash benefit payment mechanism must effectively guide the allocation of resources and leverage the human and material resources of elderly care services in order to meet the needs of the elderly (16, 17). Lack of care resources will result in resistance to the expansion of the system (18).

Huge spending on long-term care is the biggest risk elderly people facing. In the context of deepening population aging, the adoption of an ex ante financing mechanism such as LTCI is more conducive to improve policy efficiency (3). However, most countries chose ex post methods, such as public subsidies and social assistance, to finance long-term care services. People have many doubts about whether LTCI can diversify risks and reduce costs by expanding coverage, and eventually achieve the dual goals of coping with long-term care risks and financial sustainability. Only a few countries in the world have adopted the public LTCI model. China is still in the process of regional pilots. Although the Chinese government allows pilots to start with a system that only covers urban workers, they encouraged pilot cities to explore the establishment of a full-coverage system that covers all urban and rural residents (10).

The first 15 LTCI pilot cities in China draw on international experience in many aspects, especially German experience. Taking into account the fact that the contribution rate of social insurance in China is relatively high, nine out of the 15 LTCI pilot cities cover only urban workers. Five cities, including Jingmen, Shihezi, Nantong, Qingdao and Suzhou, stipulate that the target coverage groups including urban workers and urban and rural residents. Nantong is one of the three “early birds” (Nantong, Qingdao and Changchun) which have introduced policies and started a LTCI trial even before the implementation of the national pilot program. Among the three “early birds”, Qingdao was the first to explore the LTCI system in 2012, but it is limited to the scope of medical care and does not include the living care of the disabled. Changchun started exploring in 2014, but its system only covers urban workers (2). According to the field interview with the deputy director of Nantong Medical Insurance Bureau in July 2017, all other 14 pilot cities have visited and studied the Nantong model before formulating and improving their pilot policies. Jingmen, Shangrao, and Suzhou obviously borrowed the full coverage concept of Nantong (19). Since 2020, with the advancement of the second batch of LTCI pilot projects in China, the system concept of Nantong has been used for reference by more pilot cities.

Many literatures have studied the coverage effects of different pilot insurance policies (19, 20), but lack of in-depth exploration of policy innovation concepts in a single pilot area, so it is difficult to trace the institutional logic behind the coverage effects. At the same time, the existing case studies ignore the characteristic innovation indicators introduced based on regional characteristics in the calculation of long-term financial revenue and expenditure, thus affecting the accuracy of long-term financial revenue and expenditure forecasting. In the context of other regions choosing to cover only specific age groups, Nantong has taken the lead in establishing a LTCI system covering the entire population. What are the logic and reasons behind it? Are there lessons for other regions? How did the city achieve institutional financial sustainability through institutional innovation, without subsidies from the central and provincial governments? There is no existing scholarly literature addressing these questions. This article seeks to offer solutions to the above problems. Based on micro survey data, it further aims to evaluate the financial sustainability of the LTCI system in Nantong.

## Background, content, and characteristics of LTCI in Nantong

### Background: The stress of multiple disadvantages

#### Increases in deep aging and life expectancy

Nantong became an aging society in 1983, 17 years earlier than the whole country. In 2020, the city's resident population was 7.727 million, and the elderly population aged 60 and above reached 2.319 million, accounting for 30.01% of the city's population, 11.3% points higher than the national average (21). Nantong is a famous longevity city in China. At the end of 2019, the average life expectancy reached 82.61 years, 5.3 years higher than the national average. There were 400,000 elderly people aged 80 and above, accounting for 17.2% of the elderly population (22). This scenario is the result of economic and social progress, but it also brings about serious shortages in care services, along with financial burdens.

#### Low birthrates and population outflows

Nantong is experiencing decreasing birthrates and a population exodus. Since the implementation of the family planning policy, the cumulative one-child rate in Nantong has been as high as 78.6% (the highest in Jiangsu Province). In 2015, among the resident population of Nantong, there were 780,300 children aged 0-14 years, accounting for 10.69% of the total population, considerably lower than the 15% standard for “ultra-low” birthrates (23). After the implementation of the universal two-child policy, although birthrates increased in 2016 and 2017, it still did not change the overall trend. In addition, Nantong is adjacent to economically developed cities such as Shanghai and Suzhou. The pattern of population flow, in which outflows are greater than inflows, has persisted for 30 years. In 2019, the resident population was 280,000 less than the registered population (24). Most of the outflows consist of young, economically active populations and students studying abroad, resulting in the prevalence of empty-nest families and the elderly living alone. According to data provided by the Nantong Municipal Aging Office, in October 2014, there were 980,000 empty-nesters in the city, including 560,000 rural empty-nesters, accounting for about 48% of the rural elderly population and 420,000 urban empty-nesters, accounting for about 54% of the urban elderly population (25).

#### The weakening of family care and shortages in social care services

Family care is the primary form of long-term care in Nantong, but as the fertility rate continues to decline, the size of the family also continues to decline, and thus, the function of family care is weakened. In 2019, the average household size of the city's permanent resident population was 2.9, with the largest proportion of two-person and three-person households. The nuclear family has become the mainstream model (24). In addition, female employment has gradually become the norm, and the professional role of women comes into conflict with the role of caregivers that they originally played within the family. There is a serious shortage of social care service resources. In 2016, there were only six nursing homes in Nantong, with a total of less than 1,000 beds. In 2020, the number of homes had increased to 254. However, compared with Qingdao, which is also a pilot city for LTCI (with more than 850 nursing homes), nursing resources in Nantong are seriously insufficient (26). Difficulties such as the miniaturization of family size, the increase in the female employment rate, and the shortage of social care service supply have pushed Nantong to innovate the design of the social insurance system and leverage the service supply, in order to realize its long-term financial sustainability.

### Main contents and design considerations

#### Establishing a full coverage scheme through government subsidies and fallback guarantees

With its goal of “full coverage and basic protection,” the Nantong LTCI system adheres to the attributes of social insurance and the principle of government leadership, creating a model for universal participation. The nature of social insurance requires a large enough number of participants. Therefore, the Opinions on Establishing a Basic Care Insurance System (Trial) (Tong Zheng Fa [2015] No. 73) stipulates that all employees and residents, whether in urban or rural areas, must participate, in accordance with the law. In terms of specific operations, an automatic joining mechanism is introduced, and those who participate in employee medical insurance and resident medical insurance will automatically join in LTCI. Employee contributions are automatically deducted from the medical insurance personal account, and resident contributions are transferred from the medical insurance fund after the annual medical insurance premiums are collected. Minors, students, families with the minimum living allowance, families with extremely poor workers, and persons with severe disabilities in grades 1 and 2 are fully subsidized by the government. This type of government responsibility model–“paying the bottom line”–is an innovation of the Nantong pilot. Compared with other places where employees or people over 40 years old are included in the insurance coverage, the risk of disability can be spread out over the entire life cycle. Through on-the-spot interviews, this research project found several reasons for the innovation of Nantong's LTCI policy.

Compared with other social insurance programs, LTCI has certain characteristics, including fewer beneficiaries, lower risk occurrence probability, and late risk occurrence. But for families at risk, it is often the case that if one person is disabled,the whole family will be affected (27, 28). Nantong began to explore the long-term care system in the form of family beds in 2000. In 2012, it issued management measures for designated nursing homes, which included nursing homes in the scope of designated medical insurance. During the exploration, it was found that the traditional social insurance reform path of dividing the participating populations, first the urban and then the rural, could not meet the demand; instead, mandating that all residents participate would help to diversify risks and dilute costs.

We are probably the region with the most serious problem of growing old before getting rich, and the international experience of participating in insurance as an adult does not apply. Nantong's LTCI creatively established a compulsory, universal, whole-life insurance system. This is both an option and an attempt to spread the cost of disability care over a wider range and over a longer period of time. On the one hand, the system design of universal coverage prevents the risk of “fragmentation” of the system, and on the other hand, it is also an effective method to ensure the financial sustainability of the basic care insurance system under the coexistence of the deep aging of the population and ultra-low birthrates. (Respondent, government representative of the major research project “Nantong Nursing Insurance System Research” in 2013, interviewed on July 7, 2016).

#### Multi-channel financing for sharing the contribution burden

Considering that the proportion of existing social insurance contributions to personal income is already high, the Nantong LTCI pilot fundraising implements a fixed payment at a relatively low standard, tentatively set at 100 yuan per person per year, equivalent to about 3% of the per capita disposable income of urban residents in the previous year. In terms of financing methods, other pilot areas mainly rely on the allocation of medical insurance funds, while Nantong has established a sharing financing mechanism encompassing “government subsidies + personal payment + medical insurance pooling fund allocation + welfare lottery public welfare fund allocation”.

Government subsidies play a role in motivation and fallback guarantees. The government pays full contributions for certain groups of people, including minors, students, families living on subsistence allowances, employees in extreme poverty, and severely disabled persons. For others, the government subsidizes 40 Yuan for each person annually.

Specifying the proportion of government subsidies in the form of pre-financial responsibility, and implementing full subsidies to groups with special difficulties and people without income, is not only conducive to the self-balancing and financial sustainability of the care fund, but also avoids the risk of social problems based on “forcing fiscal expenditures.” It can also leverage the motivation of individuals and families to share financial resources. People understand the possible catastrophic burden of disability on individuals and families, and what is lacking is the institutional supply to deal with this risk (Respondent, government representative of the major research project “Nantong Nursing Insurance System Research” interviewed on July 7, 2016).

Individual contributions reflect the individual and family responsibilities concerning social insurance. Employee medical insurance and resident medical insurance pay 30 Yuan per person annually. The former is allocated from the individual medical insurance account, and the latter is allocated from the medical insurance payment on a per capita basis.

This is an advance preparation mechanism in the case of growing old before getting rich. It is also based on the realistic consideration that Nantong is an economically developed area and it is difficult to obtain special subsidies from the central and provincial governments. It should be noted that, unlike Qingdao and other cities that use LTCI as a part of medical insurance, Nantong's LTCI is an independent program. The logic of transferring funds from individual medical insurance involves the use of the collection channels for medical insurance to facilitate the contribution. (Respondent, deputy director in charge of LTCI, Medical Insurance Bureau, interviewed on May 15, 2017).

With regard to medical insurance fund transfers and disbursement channels, 30 Yuan per person annually is allocated from the employee medical insurance and resident medical insurance pooling funds, but it should be noted that the allocation of funds from the medical insurance fund does not mean that LTCI is a part of medical insurance.

LTCI covers medical care, which is conducive to reducing unnecessary nursing hospitalizations and reducing medical insurance expenses, the basis for the allocation of funds from medical insurance. However, Nantong's LTCI also covers life care risks and actively promotes home life care. Therefore, the municipal government has decided to gradually reduce the allocation of the medical insurance pooling fund and finally achieve the long-term goal of not being allocated from the medical insurance fund. In the future, LTCI will comprehensively consider the coverage risks to formulate separate contribution rates, and the allocation of medical insurance funds is only a transitional measure (Respondent, representative of the medical insurance department of the Medical Insurance Bureau, interviewed on May 15, 2017).

There is no clear allocation mechanism for the welfare lottery public welfare fund:

Looking at the trend, the current government not only bears the subsidy of 40 Yuan for those with income, but also bears the burden of 100 Yuan for those in difficulty and those without income. The financial investment has accounted for 47.09% of the total amount of LTCI funds. As the system matures, financial subsidies will also decrease, and the shortfall may be made up by welfare lottery funds and funds donated by social groups and individuals (Respondent, representative of civil affairs departments, interviewed on May 15, 2017).

#### Improving social acceptability by innovating the method of payment

The Nantong LTCI plan has expanded the coverage of the population and the scope of protection items. This institutional innovation improves the social acceptance of the universal coverage system and alleviates the problem of low willingness to participate in insurance, often caused by the low probability of long-term disability risk. First, Nantong's LTCI covers all persons with moderate and severe disabilities due to old age, disease, and disability, as long as their score on the Assessment Scale for Activities of Daily Living (the Barthel Index Assessment Scale) is lower than 40. Additionally, after no less than 6 months of treatment, insured persons who cannot take care of themselves and need long-term care can enjoy corresponding benefits. Second, the scope of payment is extended to living care projects. When receiving services in designated hospitals, nursing homes, and other institutions, bed fees, nursing equipment usage fees, nursing consumables fees, and nursing service fees are all included in the reimbursement catalog. Third, there is no threshold for treatment benefits, but differentiated reimbursement ratios are implemented. The reimbursement rates in designated service institutions are as follows: for those who accept bed care services from medical institutions, the reimbursement rate is 60% of the expenses within the scope of the catalog. The reimbursement rate is 50% for those who receive bed care services from elderly care institutions. Fourth, the cap payment terms are designed for door-to-door care services. Considering the reality of the lack of socialized care services in Nantong, LTCI breaks through the function of insurance that only deals with large expenditure risks. For those who receive home care services, care subsidies are issued at a minimum standard of 15 Yuan per person per day, issued on a quarterly basis. In order to overcome the risk of information asymmetry and control the scale of expenditure, the upper limit of fixed payment is 1,200 Yuan per month.

The logic of the LTCI design is to optimize treatment to motivate people to participate in insurance, and to use full coverage as a means to dilute expenses, lengthen payment time, and ultimately achieve financial sustainability of the system. Although quasi-compulsory measures such as automatic membership have been undertaken in the payment of insurance premiums, if more people cannot utilize the benefits, social acceptance will be very low. Therefore, we do not set a threshold and expand the scope of LTCI reimbursement to living care, thereby increasing the incentive to participate. The fixed payment terms are our measures to promote the development of elderly care services with insurance payment based on the provision of care services. Through fixed payment, the purchasing power of beneficiaries' care services can be improved, which is conducive to cultivating service providers. At the same time, in the case of a shortage of service supply, it can also improve people's recognition of the system. As for the lack of supervision of fixed payments, which may lead to the expansion of fund expenditures, we use strict disability assessment to control (Respondent, deputy director in charge of LTCI, Medical Insurance Bureau, interviewed on May 15, 2017).

#### Enhancing delivery capacity by introducing market forces

The social security administration agency is responsible for LTCI fund collection, guidance and supervision, and authorizes third parties to operate insurance service businesses under the principle of obtaining marginal profits. Currently, the third parties include an insurance corporate body composed of four insurance companies: PingAn Pension, TaiPing Pension, Pacific Life, and China Life Insurance Company. The corporate body operates the above businesses with a management fee rate of 1–3% from LTCI fund. If the overrun is <5%, it shall be borne by the third party (co-insurer). Overruns of 5–10% shall be shared by the government and the third party. By utilizing third party technology and network advantages, management costs can be reduced.

The Human Resources and Social Security Department is responsible for fundraising, handling business guidance, service agency determination and business supervision, purchasing services through the government, and entrusting third-party agencies to participate in the handling in accordance with the principle of maintaining capital and small profits. The specific handling businesses include acceptance assessment, fee review, operation payment, nursing staff capacity development, service supervision, audit investigation, and information maintenance, among others.

#### Cultivating service entities through insurance payment mechanisms

The LTCI agency formulates the access standards for agency service entities, and implements agreement management for designated service agencies that meet the standards. In order to promote competition, the agreement is signed annually. The payment model of the institution is based on the total headcount, and there are three types of treatment payment standards. First, those who receive nursing services in nursing beds located in medical institutions can also enjoy basic medical insurance inpatient treatment in those institutions. Persons with severe disability and dementia are paid by the care insurance fund at the rate of 70 Yuan per person per day, and those with moderate disabilities are paid at the rate of 50 Yuan per person per day. Second, those who receive nursing services in nursing beds located in old-age service institutions will be paid by the nursing insurance fund at a rate of 50 Yuan per person per day for severely disabled persons, and 40 Yuan per person per day for moderately disabled persons. Third, for disabled persons who have registered poor families and family members with minimum subsistence allowances, and receive care services in care institutions, the treatment payment standard will be increased by 20%.

Expenses within the scope of the LTCI service catalog are subject to a pro-rata reimbursement policy for beneficiaries, but service agencies are not paid by item, but rather by the total headcount. The fixed payment standard is formulated according to market research, which is conducive to guiding service providers to provide basic services, and preventing the provision of services induced to exceed the standard under the project-based payment mechanism. At the same time, the total settlement only involves fees within the scope of the LTCI service catalog. This provision does not restrict the freedom of service providers to provide individuals with personalized services outside the catalog and the cost of self-payment of service standards for individuals. Nursing homes are also graded and the price of each is different. In Nantong, inexpensive homes are 2000-3000 Yuan/month, and the high-end homes run about 7000-8000 Yuan/month. The upper limit of the subsidy is 1,500 Yuan. If your economic conditions are good, you can choose a high-end one and pay a little more, or choose an inexpensive one if you have financial difficulties; then, you need to pay about 500-600 Yuan. This reflects the basic principle of LTCI and is also conducive to the cultivation of multi-level markets. (Respondent, director of LTCI Fund Supervision Department, interviewed on May 15, 2017).

#### Encouraging acceptance of home service to reduce service costs

Accepting door-to-door care services at home is not only in line with the actual situation of Chinese elderly people, often unwilling to leave home, but it can also overcome the problem of insufficient supply of institutional service hardware (beds) in the initial stages of LTCI implementation. LTCI plans to actively promote home-based services while introducing cash subsidies. At present, a total of six door-to-door packages have been developed: two “Ankang” packages and four “Hukang” packages. The price ranges from 300 to 500 Yuan per cycle. Every four door-to-door services constitute a cycle, but only for severely disabled people. The cost of home care services accepted by the package will be borne by the individual and the fund, paying 10 and 90%, respectively. Formal care at home is not only what most disabled people need, but also represents an effective way to reduce the cost of care expenditure.

## Evaluation of the financial sustainability of LTCI in Nantong

### A financial balance estimation model for LTCI

Based on the EU's LTCI revenue and expenditure forecasting framework (11), this study incorporates Nantong's socioeconomic and LTCI system characteristic indicators to establish a financial prediction model that will evaluate the effectiveness of the innovation of Nantong's LTCI system from the perspective of financial sustainability.

#### Model construction

##### Annual revenue of LTCI

The financing standard of Nantong's LTCI is determined based on per capita disposable income, so the factors affecting the total annual income of LTCI include the total population, financing standards, and per capita disposable income. The formula is expressed as follows:


(1) $$\begin{array}{l} R_{\text{t}}=y_{t}\times s_{{}_{t}}\times N_{t}=\left(y_{t}\times N_{t}/GDP_{t}\right)\times \left(GDP_{t}/N_{t}\right)\\ \;\times s_{t}\times N_{t} \end{array}$$

Among the components, *R*_t_ is the annual revenue of LTCI, y_*t*_ is per capita disposable income, *s*_*t*_ is the contribution ratio, and*N*_*t*_is the total population in year t.

Further, the proportion of the annual revenue of LTCI to GDP can be expressed by the product of disposable income as a share of GDP and the contribution ratio. The equation is structured as follows:


(2) $$\begin{array}{l} R_{t}/GDP_{t}=\left(y_{t}\times N_{t}\right)/GDP_{t}\times s_{t} \end{array}$$

##### Annual expenditure of LTCI

Factors affecting the total annual expenditure on LTCI include the total population, the disability rate, and the institutional and home care ratios and their corresponding costs. The formula is calculated as follows:


(3) $$\begin{array}{l} E_{t}=\left(N_{t}^{0-59}d_{t}^{0-59}+\overset{21}{\underset{x=13}{\sum }}N_{t}^{x}d_{t}^{x}\right)\times \left[p_{i}e_{i}+\left(1-p_{i}\right)e_{h}\right] \end{array}$$

*E*_*t*_ represents the annual expenditure on LTCI; $N_{t}^{x}$ and $d_{t}^{x}$ represent the population total and the disability rate in year t and *x* age, respectively; $d_{t}^{0-59}$and $N_{t}^{0-59}$ represent the disability rate and the number of people aged 0-59 years, respectively; *p*_*i*_ and (1−*p*_*i*_) represent the institutional care ratio and home care ratio, respectively; *e*_*i*_ and *e*_*h*_ represent annual per capita expenditure for institutional care and family care, respectively.

#### Parameter setting and calculation of the main variables

The calculation of revenue and expenditure for LTCI should be based on detailed demographic data, including analyses based on age-specific population data. This study combines the population data of Nantong from the Jiangsu Province 2010 Census Data, the Life Table of China's Life Insurance Industry (2010-2013), and the Nantong Statistical Yearbook 2010-2020 to determine the relevant parameters and predict the revenue and expenditure data for LTCI in Nantong.

##### Total population calculation

The total population calculation consists of three steps, calculating first the birth rate, second, the mortality rate, and then, third, the total population and age-specific population data are calculated based on the Nantong census data, combined with the birth rate and mortality rate.

###### Birth rate

The birth rate is the ratio of the number of babies born in a certain area to the total number of babies in a certain period of time (usually one year), expressed in thousandths. First, the birth rate data for 1990-2018 in Nantong, released by the Nantong Municipal Bureau of Statistics, are fit. The fitted birth rate equation reads as follows:


(4) $$\begin{array}{l} {\text{f}}_{t}=0.1173t+5.8015\left(t=1,2,3....29\right) \end{array}$$

Then, the birth rate after 2018 is extrapolated according to the fitted birth rate formula, and when the upper limit of the birth rate corresponding to the assumed total fertility rate is reached, it will not change. The formula for calculating the upper limit of the birth rate is:

$br_{t}=TFR\times N_{ft}^{15-49}/N_{t}/35\times 1000$, in which, *br*_*t*_ indicates the birth rate, *TFR* indicates the total fertility rate, *N*_*t*_ indicates the total population, and $N_{ft}^{15-49}$ indicates the number of women of childbearing age. Considering the underreporting in the census and the further improvement of the future birth policy, the total fertility rate is slightly increased, based on the 2021 census data (1.3), combined with the total fertility rate warning line (1.8) and the total fertility rate meeting the normal population replacement (2.1), the total fertility rate of women of childbearing age in Nantong can reach four scenarios: low (1.4), medium low (1.6), medium high (1.8), and high (2.1), Four upper limits of the birth rates are obtained accordingly, They are low (8%), medium low (9%), medium high (11.18%) and high (13.04%) respectively.

###### Mortality

Because the census data take a five-year increment as an age group, this article also uses five-year increments to calculate the arithmetic average of the mortality rate among the corresponding age group on the basis of China's Life Insurance Industry Experience Life Table (2010-2013). Population empirical mortality rates are formed for each age group.

###### Calculation of the total population of each age stage from 2020 to 2110

First, based on the actual birth population data of Nantong from 2010 to 2020 and the results of the birth rate and death rate calculations, the population of 1 to 4-year-old persons in 2015 and 2020 are calculated. Combined with the 2010 Nantong census data, the population age shift algorithm is used to calculate the population data of 5 to 9-year-old persons and later in Nantong in 2020. Further, on this basis, the change trend of the total population and structure of Nantong from 2020 to 2110 is predicted.^3^

The first step is to predict the 0-year-old population. The 0-year-old population is the basis for the prediction of the age shift algorithm. The 0-year-old population in 2020 is the actual birth population of Nantong in that year multiplied by the 0-year-old survival rate (represented by [1-death rate]), and the 0-year-old population in subsequent years is predicted based on the 2020 Nantong population:

$N_{t}^{0}=N_{t-1}\times br_{t}\times \left(1-dr^{0}\right)$, in which, $N_{t}^{0}$ and *br*_*t*_ indicate the number of people aged 0 and the birth rate in year t, respectively, *N*_*t*−1_ indicates the total population for the last five years, and *dr*^0^ indicates the 0-year mortality rate.^4^

The second step is to calculate the population aged 1-4 years by using the equation:


(5) $$\begin{array}{l} N_{\text{t}}^{1-4}=\overset{4}{\underset{m=1}{\sum }}N_{t-1}\times br_{t-m}\times \left(1-\text{d}{\text{r}}^{1-4}\right) \end{array}$$

In Step 3, the population number of people aged 0-4 years old is calculated. The number of people aged 0–4 years is obtained by summing the first two steps.

In Step 4, age shift is calculated. If the population of a certain age group in a five-year increment is multiplied by the survival rate of the next age group, we obtain the population of the next age group in the next five-year increment. The calculation formula is $N_{t+1}^{x+1}=N_{\text{t}}^{x}\times \left(1-br^{x+1}\right)$. By doing this, we can gradually calculate the population for each five year increment.

The fifth step is to use the above method to estimate until the year 2110, in five-year increments. According to the assumptions in this study, there are four scenarios for the expected realization of the total fertility rate and the corresponding upper limit of the total population birth rate, so that four different groups of population data, by age group, for the five-year increments from 2020 to 2110, can be obtained.

##### Calculation of the disability rate

###### Disability rate of the population aged 59 and below

By the end of 2019, Nantong had 7.5 million people covered by LTCI, 30.8% of the population over the age of 60, and 25,727 people enjoying benefits, of which 8.6% were aged 59 and below. Based on this, it can be calculated that the disability rate of the population aged 59 and below is 0.4%.^5^

###### Disability rate of the elderly aged 60 and above

Due to the lack of data on disability rates by age group in Nantong, the rates for the inability for people to take care of themselves by age group in the Jiangsu Province 2010 Census Data are used instead. Disability rate by age group equals the number of elderly people who cannot take care of themselves in a certain age group divided by the total number of survey samples of the elderly in this age group. For the convenience of calculation, it is assumed that the disability rate is only related to age, that is, the disability rate of a certain age group in 2010 is the same as that in 2110, thus forming the distribution of the disability rate in each age group.

##### The ratio of institutional care to home care

Since the implementation of the LTCI system in Nantong, the cumulative number of people enjoying benefits is 25,727 (21), of which 21,640 are at home, 3,296 are in nursing institutions, and 791 are in nursing homes. Based on this, it is estimated that institutional care accounts for 16%, which is much lower than the levels in other countries (26). In 2008, the proportion of institutional care in Germany, the Netherlands, Japan, and South Korea was 32, 39, 22, and 50%, respectively (29). There is still much of room for improvement in the proportion of institutional care in Nantong. Taking the relatively high levels abroad as a reference, it is assumed that the proportion of institutional care will increase by 1% point every 2 years on the basis of 16% in 2019, and will remain unchanged once it reaches 30%. The distribution in the proportions of institutional care and home care in Nantong from 2020 to 2110 can be calculated.

###### Annual per capita fund expenditure for home care and institutional care

First, the annual per capita expenditure of the care fund is calculated. The formula for calculating the annual per capita fund expenditure for each type of care service is as follows: *e*_*x*_ = *S*_*Bx*_ × 365 (*e*_*x*_indicating the annual per capita expenditure of the care fund, *S*_*Bx*_ indicating the standard of treatment per person per day). Due to the different treatment standards in home care versus institutional care, institutions are further divided into nursing institutions and nursing homes, and the standards are also different. The fixed settlement standards for nursing institutions and nursing homes are 70 Yuan and 50 Yuan per person per day, respectively, and the cash subsidy is paid at a fixed rate of 15 Yuan per person per day for home care. The LTCI fund settlement standard for choosing an elderly care service package refers to the cash subsidy. The annual per capita expenditure of the three types of care can be calculated according to the above-mentioned formula for the per capita expenditure of the fund, and, then, the annual per capita expenditure of the institutional care fund can be calculated by taking the proportion of patients cared for by the two institutions as the weight. It is estimated that the annual per capita fund expenditure of households and institutions is 5,475 Yuan and 24,137 Yuan respectively.

Then, the annual per capita spending of the Care Fund for 2020–2110 is forecasted. Since the main portion of long-term care costs consists of labor costs, it is assumed that the growth rate in fund expenditures for institutional and home care is roughly in line with the growth rate in average per capita social wages. First, based on the social average wage growth rate of 6.8% from 2015 to 2019 (24), economic growth is classified as “high” (the average wage growth rate drops by 1% point every 10 years until it drops to 2.4% and then remains unchanged) (29) and “low” (the average wage growth rate drops by 1% point every 5 years until it drops to 2.4% and remains unchanged); the wage growth rate from 2020 to 2110 is forecasted under the corresponding scenario. Then, based on the annual per capita expenditure of the LTCI fund in 2020, the per capita expenditure of the home and institutional LTCI fund from 2020 to 2110 is forecasted according to the same trend as the wage growth rate.

###### Proportion of urban residents' disposable income to GDP

From 2015 to 2019, the average annual per capita disposable income of Nantong residents accounted for 39% of per capita GDP (24), which is still far behind the level of 60–70% in developed countries. In recent years, however, as economic development has continued to focus on people's livelihood, the proportion of Chinese residents' disposable income in GDP has begun to rise. Considering that the per capita GDP level is relatively low, the proportion of disposable income remains low in order to achieve a relatively high economic growth rate. This study sets four capping scenarios before 2110, in which the proportion of disposable income in GDP does not exceed 45, 50, 55, and 60%, respectively. It is assumed that the proportion of Nantong's disposable income to GDP will increase by 1% point every 2 years on the basis of 2019, and will remain unchanged after it reaches the ceiling. Thus, the upper limit of the proportion of disposable income in GDP is 45% (A), 50% (B), 55% (C) and 60% (D). The data on the proportion of disposable income in GDP from 2020 to 2110 are thus formed.

#### Calculation results

##### Proportion of total LTCI expenditure to GDP

According to the previous formulas and calculation results, two scenarios, with high and low growth rates, can be calculated (assuming growth rates of 1% point per 10-year decline and 1% point per 5-year decline from 2019 levels, until reaching 2.4%) and from 2020 to 2100, the groups of women of childbearing age include: low (1.4), medium low (1.6), medium (1.6), medium high (1.8) and high (2.1), respectively, forming eight groups of LTCI expenditure in GDP proportion data.

It can be seen from Table 1 that under the current LTCI system in Nantong, the total expenditure scale of the fund is well controlled, reflecting the system's design concept. Even in the year when the total expenditure on LTCI as a share of GDP peaks (2055), the share is only about 0.33%, lower than that of other countries with insurance models, except for South Korea (29). This scenario is mainly attributable to the promotion of home care with fixed subsidies, and the capping and settlement of institutional care to force care institutions to move from treatment to health maintenance.

**Table 1:** Proportion of total LTCI expenditure in GDP in Nantong from 2020-2110 under different economy-population scenarios (%).

**Year**	High *high	High *middle high	High *middle and low	High *low	Low *high	Low *medium high	Low *medium and low	Low *low
2020	0.0848	0.0869	0.0863	0.0863	0.0848	0.0869	0.0863	0.0863
2025	0.1288	0.1301	0.1294	0.1294	0.1290	0.1302	0.1295	0.1295
2030	0.1727	0.1729	0.1727	0.1727	0.1730	0.1733	0.1730	0.1730
2035	0.2153	0.2153	0.2153	0.2152	0.2159	0.2159	0.2159	0.2159
2040	0.2595	0.2595	0.2595	0.2594	0.2605	0.2605	0.2605	0.2604
2045	0.3040	0.3040	0.3040	0.3039	0.3055	0.3055	0.3055	0.3054
2050	0.3287	0.3287	0.3286	0.3285	0.3306	0.3306	0.3305	0.3304
2055	0.3321	0.3321	0.3320	0.3319	0.3344	0.3344	0.3343	0.3342
2060	0.3293	0.3293	0.3292	0.3290	0.3319	0.3318	0.3317	0.3316
2065	0.3014	0.3013	0.3012	0.3010	0.3040	0.3040	0.3038	0.3036
2070	0.2510	0.2508	0.2507	0.2505	0.2534	0.2533	0.2531	0.2529
2075	0.2195	0.2194	0.2192	0.2189	0.2219	0.2217	0.2215	0.2213
2080	0.2088	0.2086	0.2084	0.2081	0.2111	0.2109	0.2107	0.2104
2085	0.2024	0.2022	0.2019	0.2016	0.2046	0.2044	0.2041	0.2038
2090	0.1806	0.1803	0.1801	0.1796	0.1826	0.1823	0.1820	0.1815
2095	0.1550	0.1547	0.1544	0.1535	0.1567	0.1564	0.1561	0.1551
2100	0.1468	0.1464	0.1460	0.1442	0.1484	0.1480	0.1476	0.1458
2105	0.1533	0.1529	0.1520	0.1488	0.1550	0.1546	0.1536	0.1504
2110	0.1663	0.1657	0.1641	0.1585	0.1681	0.1675	0.1658	0.1603

“Economy^*^Population” in the table represents a combination of scenarios. For example, “High^*^High” represents a combination of high economic growth and high population growth scenarios.

Calculations based on the 2010 Census of Jiangsu Province and online data from Nantong Statistics Bureau.

##### Proportion of total LTCI revenue to GDP

According to the current system in Nantong, the total annual income of LTCI is the product of the total population, per capita GDP, the proportion of disposable income to GDP, and the total financing ratio (3%). Total GDP is the product of population and GDP per capita. In this way, regardless of the economic growth and population growth scenarios, the proportion of total LTCI income to GDP is the product of the proportion of disposable income to GDP and the total financing ratio (3%). ^6^

**Figure 1:**
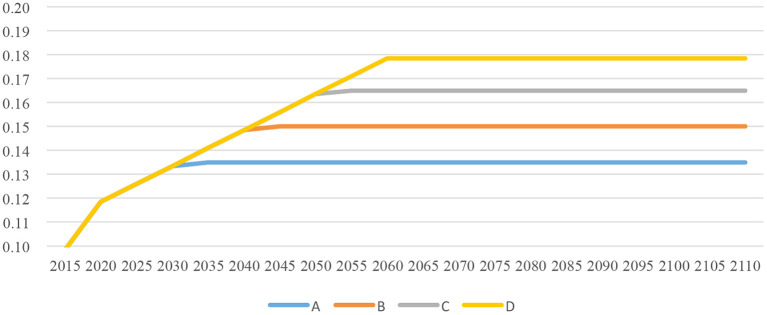
The proportion of LTCI income in GDP under the four scenarios (%).

According to the above formula and calculation results, total income as a percentage of GDP data can be calculated when the proportion of disposable income in GDP reaches the upper limit of 45% (A), 50% (B), 55% (C) and 60% (D), respectively (see Figure 1). As can be seen from Figure 1, assuming that the current system remains unchanged, in the long run, the proportion of LTCI income to GDP falls between 0.1 and 0.18%. The more the distribution of national income skews toward disposable income, the higher the total income for LTCI.

##### Calculation of the current revenue and expenditure gap of LTCI

The current income of the LTCI fund is affected by the four scenarios of disposable income proportion (A, B, C, D), and the current expenditure is affected by the high and low economic growth scenarios and the low, medium-low, medium-high, and high population growth scenarios Therefore, 32 combinations of current balance of payments gaps are formed. Tables 2 , 3 report the year in which the deficit occurred under the 32 scenarios, the year in which the deficit accounted for the highest proportion of GDP in that year, the year in which the transition from deficit to surplus began, and the corresponding values.

**Table 2:** Financial deficit or surplus of the LTCI system as a percentage of GDP under the low economic growth scenario (%).

**Disposable income distribution scenario**		**Low economic growth and various combination of population growth scenarios**			
		**Low population growth**	**Middle and low population growth**	**Middle and high population growth**	**High population growth**
A	Deficit begin	Year 2025 (−0.0035)	Year 2025 (−0.0035)	Year 2025 (−0.0042)	Year 2025 (−0.0030)
	Highest deficit	Year 2055 (−0.1992)	Year 2055 (−0.1993)	Year 2055 (−0.1994)	Year 2055 (−0.1994)
	Surplus	–	–	–	–
B	Deficit begin	Year 2025 (−0.0035)	Year 2025 (−0.0035)	Year 2025 (−0.0042)	Year 2025 (−0.0030)
	Highest deficit	Year 2055 (−0.1842)	Year 2055 (−0.1843)	Year 2055 (−0.1844)	Year 2055 (−0.1844)
	Surplus	Year 2100 (0.0042)	Year 2100 (0.0024)	Year 2100 (0.0020)	Year 2100 (0.0016)
C	Deficit begin	Year 2025 (−0.0035)	Year 2025 (−0.0035)	Year 2025 (−0.0042)	Year 2025 (−0.0030)
	Highest deficit	Year 2055 (−0.1692)	Year 2055 (−0.1630)	Year 2055 (−0.1694)	Year 2055 (−0.1694)
	Surplus	Year 2095 (0.0099)	Year 2095 (0.0089)	Year 2095 (0.0086)	Year 2095 (0.0083)
D	Deficit begin	Year 2025 (−0.0035)	Year 2025 (−0.0035)	Year 2025 (−0.0042)	Year 2025 (−0.0030)
	Highest deficit	Year 2050 (−0.1669)	Year 2050 (−0.1670)	Year 2050 (−0.1671)	Year 2050 (−0.1671)
	Surplus	Year 2095 (0.0234)	Year 2095 (0.0224)	Year 2095 (0.0221)	Year 2095 (0.0218)

The figures in parentheses represent the proportion of the current deficit in GDP.

Calculations are based on the Jiangsu Province 2010 Census Data, the online data of Nantong Bureau of Statistics, and the data provided by Nantong Human Resources and Social Security Bureau.

**Table 3:** Financial deficit or surplus of the LTCI system as a percentage of GDP under the high economic growth scenario (%).

**Disposable income distribution scenario**		**High economic growth and a combination of various population growth scenarios**			
		**Low population growth**	**Middle and low population growth**	**Middle and high population growth**	**High population growth**
A	Deficit begin	Year 2025 (−0.0034)	Year 2025 (−0.0034)	Year 2025 (−0.0041)	Year 2025 (−0.0028)
	Highest deficit	Year 2055(−0.1969)	Year 2055 (−0.1970)	Year 2055 Year (−0.1971)	Year 2055 Year (−0.1971)
	Surplus	–	–	–	–
B	Deficit begin	Year 2025 (−0.0034)	Year 2025 (−0.0034)	Year 2025 (−0.0041)	Year 2025 (−0.0028)
	Highest deficit	2055 Year (−0.1819)	2055 Year (−0.1820)	2055 Year (−0.1821)	Year 2055 (−0.1821)
	Surplus	Year 2100 (0.0058)	Year 2100 (0.0040)	Year 2100 (0.0036)	Year 2100 (0.0032)
C	Deficit begin	Year 2025 (−0.0034)	Year 2025 (−0.0034)	Year 2025 (−0.0036)	Year 2025 (−0.0028)
	Highest deficit	Year 2055 (−0.1669)	Year 2055 (−0.1670)	Year 2055 (−0.1671)	Year 2055 (−0.1671)
	Surplus	Year 2095 (0.0115)	Year 2095 (0.0106)	Year 2095 (0.0103)	Year 2095 (0.010)
D	Deficit begin	Year 2025 (−0.0034)	Year 2025 (−0.0034)	Year 2025 (−0.0041)	Year 2025 (−0.0028)
	Highest deficit	Year 2050 (−0.1650)	Year 2050 (−0.1651)	Year 2050 (−0.1652)	Year 2050 (−0.1652)
	Surplus	Year 2095 (0.0250)	Year 2095 (0.0241)	Year 2095 (0.0238)	Year 2095 (0.0235)

The figures in parentheses represent the ratio of the current deficit to GDP.

Calculations based on the 2010 Census of Jiangsu Province, the online data of Nantong Statistics Bureau and the data provided by Nantong Human Resources and Social Security Bureau.

First, the gap between the current income and expenditure of Nantong's LTCI has been well controlled. Starting in 2025, the current balance of payments will start to show a deficit in all scenarios, and it will continue to increase and peak over the next 25–30 years, but the scale is not large. The highest scenario is a combination of low disposable income distribution, high economic growth, and high population growth, with the current deficit as a percentage of GDP peaking at 0.1971% in 2055.

Second, the share of disposable income is the key to affecting the current income and expenditure of LTCI. Since the financing standard of Nantong's LTCI is determined based on per capita disposable income, the more inclined the income distribution is to individuals, the more conducive it is to reduce the proportion of the current deficit to GDP in the year with the highest deficit. At the same time, it is more conducive to realizing the current surplus earlier. It can be seen from Tables 2 , 3 that as long as the disposable income accounts for a low proportion of GDP in Scenario A, regardless of the combination of the economic growth and population growth scenarios, there will be no surplus before 2110. If disposable income increases as a share of GDP in scenario B, a surplus occurs in 2100. If the proportion of disposable income in GDP is further increased to scenarios C and D, the current balance of payments will be surplus in 2095.

Third, although high economic growth cannot eliminate deficits and delay their appearance, it can reduce the size of deficits. As can be seen from Tables 2 , 3, other scenarios are the same, the annual deficit in the high economic growth scenario is lower than that in the low economic growth scenario.

Fourth, due to the long time lag between birthrate changes and demographic changes, in the 2020-2110 measurement period, a high population growth rate may actually increase the ratio of LTCI revenue to GDP in the year with the highest deficit (see Tables 2, 3).

##### Calculation of the depletion year and the cumulative gap scale of the LTCI fund

It can be seen from the calculation of current revenue and expenditure that all scenarios from 2015 to 2025 can maintain the current surplus. This implies that the LTCI system is likely to reach a peak cumulative surplus of funds around 2025. At that point, with the appearance and increase of the current balance of payments deficit, the accumulated capital surplus will decrease until it is exhausted, and a cumulative gap will appear, reaching the peak of the cumulative gap around 2060. The gap then gradually narrows and a cumulative surplus emerges around 2095.

The following is an example of the scenario in which disposable income eventually stabilizes at 55% of GDP (Scenario C) and the total fertility rate stabilizes at 1.8 (mid-to-high fertility) to calculate the year and cumulative gap in the cumulative gap of LTCI funds under high and low economic growth scenarios percentage of GDP for that year. Table 4 shows that under the two economic growth scenarios, the capital pool formed by the accumulation of current surpluses will be exhausted by 2030. There will be a fund accumulation gap in 2030, and the gap will reach its peak in 2060 (accounting for 0.83-0.85% of GDP in that year), and by 2110, the fund accumulation will turn from a gap into a surplus. Compared with high economic growth, the scale of low economic growth gap is slightly larger (about 0.84%) (see Table 4). In general, although there is a cumulative gap in the LTCI foundation under the current system, the scale is not large, and it is within the range of personal and financial affordability.

**Table 4:** Percentage of the accumulated amount of LTCI funds in GDP under the combination of income distribution scenario C and total fertility rate 1.8 scenario (%).

**Year**	**High economic growth**	**Low economic growth**
2020	0.4138	0.4138
2025	0.1225	0.1223
2030	−0.0558	−0.0567
2035	−0.2319	−0.2340
2040	−0.4080	−0.4116
2045	−0.5920	−0.5975
2050	−0.7573	−0.7652
2055	−0.8277	−0.8377
2060	−0.8327	−0.8443
2065	−0.7934	−0.8062
2070	−0.6311	−0.6441
2075	−0.3977	−0.4098
2080	−0.2610	−0.2727
2085	−0.2116	−0.2229
2090	−0.1640	−0.1747
2095	−0.0510	−0.0606
2100	0.0596	0.0513
2105	0.0863	0.0783
2110	0.0477	0.0392

Calculated according to the Jiangsu Province 2010 Census Data, the online data of the Nantong Bureau of Statistics, and the data provided by Nantong Human Resources and Social Security Bureau.

## Limitations

This article has examined the institutional logic of Nantong's long-term care model, designed to achieve full coverage through institutional innovation, as well as long-term financial sustainability. Due to the space limitations, it does not discuss possible improvements to the Nantong model. For instance, in Nantong's current LTCI financing channel, the allocation ratio of the basic medical insurance pooling fund is 30%, but the planning goal of the government is to gradually reduce the financing ratio of this channel until it is entirely canceled. Whether the financing channels of basic medical insurance should be improved or canceled warrants further investigation. In addition, in terms of welfare lottery financing channels, Nantong must institutionalize and clarify the amount or proportion of public welfare financial funds. Specific measures to do so should be explored in further research (30). Further, to this point, the system has only been implemented for a limited time, the actual number of people aged 0–59 who enjoy the treatment is relatively low, so the estimated disability rate under age 60 may be biased.

## Conclusion

Through field interviews with officials and experts involved in implementing the Nantong LTCI model, this research has examined the LTCI system in Nantong, exploring its logic and methods for successfully realizing institutional innovation. The study further uses quantitative methods to measure the system's financial sustainability. According to the calculated results, under all hypothetical scenarios, the current and cumulative LTCI revenues and expenditures balance comprise a “U” shape. The exception is Scenario A (in which disposable income accounts for less than 45% of GDP),with a deficit in 2025 for the first time, a peak in 2055, and a surplus in 2095-2100. Considering the fact that China's income distribution is low as a share of GDP and the government has begun to work on increasing this share as part of its long-term development goals, the probability of Scenario A is extremely low. In all scenarios, the peak current deficit is below 0.2% of GDP, and the cumulative deficit is below 0.9% of GDP. The above evaluation indicates that coverage expanding is an effective way to achieve financial sustainability.

Through horizontal and vertical two-way transfer payment, the Nantong model not only achieves full coverage, but also makes an important contribution to financial sustainability. The traditional LTCI system, which only covers certain age groups, only realizes horizontal transfer payments between people with different nursing needs in the same generation. The Nantong model not only includes this traditional horizontal transfer payment, but also realizes intra-generational vertical transfer payments for individuals in different stages of the life cycle.

Further, the study has found that through “full coverage” and “multiple financing” models, the Nantong pilot program has not only broken away from the traditional dual system and fragmented path dependence models, but has also achieved a unified system at the system's establishment. The integrated development has also changed the inherent thought framework of the traditional medical insurance system, which creates different systems for different groups of people, a result that has important theoretical and practical significance. This non-differentiated system construction method, with full coverage and the practice of multiple financing, provides valuable experience for developing countries and regions.

## Data availability statement

The original contributions presented in the study are included in the article/supplementary material, further inquiries can be directed to the corresponding author.

## Author contributions

All authors listed have made a substantial, direct, and intellectual contribution to the work and approved it for publication.

## Funding

We gratefully acknowledge the financial support from the National Social Science Foundation of China (No. 20BRK027).

## Conflict of interest

The authors declare that the research was conducted in the absence of any commercial or financial relationships that could be construed as a potential conflict of interest.

## Publisher's note

All claims expressed in this article are solely those of the authors and do not necessarily represent those of their affiliated organizations, or those of the publisher, the editors and the reviewers. Any product that may be evaluated in this article, or claim that may be made by its manufacturer, is not guaranteed or endorsed by the publisher.

## Footnotes

^1^What is the population aging standard? In which year did Nantong enter an aging society? http://www.nantong.gov.cn//ntsrmzf/cjwtjd/content/3e4dd435-1df9-4e38-be23-c9bc6650b110.html (accessed December 1, 2021).

^2^Bulletin of the Seventh National Census of Nantong (No. 4), http://tjj.nantong.gov.cn/ntstj/tjgb/content/207c7940-dff2-47d9-89dd-cd939acfe4f8.html (accessed December 1, 2021).

^3^The population age-shift algorithm takes the actual population number of each 5-year age group according to the birth rate and mortality rate of the corresponding age group, and predicts the population of each age group in the corresponding five years.

^4^Because the data is calculated every 5 years, the same population within five years. In other words, assume that the first four years of change are concentrated in the fifth year.

^5^Disability rate for people aged 59 years and below.$d^{0-59}=\frac{25727\times 8.6\%}{7500000\times \left(1-30.8\%\right)}$.

^6^Nantong's LTCI financing standard is 100 yuan per person. Per year 40 yuan is raised from financial subsidies, 30 yuan from the medical insurance pooling fund, and individuals paid 30 yuan. The financing standard is determined by referring about 3 per thousand of the per capita disposable income of urban residents in the previous year, and the financing ratio is set at 3 percent here.
